# Antimicrobial Resistance Patterns and ESBL of Uropathogens Isolated from Adult Females in Najran Region of Saudi Arabia

**DOI:** 10.3390/clinpract11030080

**Published:** 2021-09-14

**Authors:** Mohammed Yahia Alasmary

**Affiliations:** Medical Department, College of Medicine, Najran University, Najran 1988, Saudi Arabia; alasmary31@hotmail.com

**Keywords:** urinary tract infections (UTIs), ESBL, antibiotic, *Escherichia coli*

## Abstract

Background: To explore the prevalence of urinary tract infections (UTIs) among female patients in the Najran region of Saudi Arabia and determine their antimicrobial resistance pattern. Methods: This study was conducted on 136 urine samples collected from outpatient departments (OPDs) of the different government hospitals in the Najran region of Saudi Arabia. Over one year, the results of susceptibility testing reports of outpatient midstream urine samples from three government hospitals were prospectively evaluated. Results: Of 136 urine samples, only 123 (90.45%) were found to show significant growth for UTIs, from which 23 different uropathogens were identified. *Escherichia coli* (58.5%) was the most commonly isolated organism, followed by *Klebsiella pneumoniae* (8.1%). The isolated microorganism showed increased resistance patterns from 3.3% to 62.6%, with an overall resistance of 27.19%. Meropenem was the most effective antimicrobial, followed by amikacin and ertapenem (0.47%, 0.91%, and 1.5% resistance, respectively). At the same time, ampicillin and cephazolin were the least (62.6% and 59.5% resistance, respectively) effective. Overall, eleven (8.94%) uropathogens isolates were ESBLs, among which there were eight (6.5%) *Escherichia coli*, one (0.81%) *Klebsiella pneumoniae*, one (0.81%) *Klebsiella oxytoca*, and one (0.81%) *Citrobacter amalonaticus*. Conclusions: *E. coli* remains the most commonly isolated causative uropathogens, followed by *Klebsiella* species. The prevalence of pathogenic *E. coli* and *Klebsiella* species underscores the importance of developing cost-effective, precise, and rapid identification systems to minimize public exposure to uropathogens. Antibiotic susceptibility data revealed that most of the isolates were resistant to the majority of the antibiotics. The patients with UTIs in the Najran region of Saudi Arabia are at a high risk of antibiotic resistance, leading to significant problems in outpatient department (OPD) treatment outcomes and raising the alarm for the physician to change their empiric treatment.

## 1. Introduction

Urinary tract infections (UTIs) are the most common disease, from uncomplicated cystitis to urosepsis and septic shock [1,2]. UTIs are the most common bacterial infection encountered in clinical practice affecting people of all ages, primarily females [3,4,5,6]. As reported by the WHO, an estimated 50 % of women report having UTIs at some point in their lives [7]. It makes up a significant amount of antibiotic prescriptions, resulting in clinical and economic burdens in the community setting [2,4,6,7,8,9,10]. UTIs are mainly caused by Gram-negative bacteria, which account for 80–85%, and the most causative organism is *Escherichia coli* (50–70%), followed by *Klebsiella* [11,12].

Increasing multidrug resistance (MDR) among pathogens is of global concern. MDR in both OPD and patients with UTIs is rising and can vary according to geographical and regional location [13,14,15]. Furthermore, extended-spectrum beta-lactamase (ESBL)-producing organisms have additional therapeutic implications since they exhibit resistance to various potent antibiotics, including third-generation cephalosporin, extended-spectrum penicillin, and monobactams; cause global problems; and produce significant diagnostic and therapeutic challenges [12,16]. The prevalence of ESBL-producing bacterial strains among isolated organisms has progressively increased over the past decades [12,16]. In addition, due to cross-resistance to other antimicrobial agents, the therapeutic options in UTIs caused by ESBL-producing microbes are limited, which makes the treatment more complex and the use of broad-spectrum antibiotics expensive [17,18,19,20,21]. Because of this global rise in MDR over time, the significant difference in antimicrobial susceptibility in different geographical regions, the prevalence of the ESBL strain among clinical isolates, and the routine assessment of the antimicrobial susceptibility pattern, resistance surveillance at local, national, and international levels is of paramount importance to facilitate the selection of empiric UTIs therapy. This study was carried out to determine the prevalence of antibiotic resistance patterns, including ESBLs among the multidrug-resistant microbes isolates from females patients in the Najran region of Saudi Arabia over a 12-month period.

## 2. Methods

Reports were collected from the microbiology laboratories of three government hospitals (King Khalid hospital, Najran General hospital, and Najran University hospital) in Najran, Kingdom of Saudi Arabia, for one year from December 2019 to January 2021. Ethical approval was taken from the scientific research ethical committee (reference no 442-42-24821-DS), Najran University, Kingdom of Saudi Arabia. All patients were referred to the microbiology laboratories either by urologists or health care physicians. The patients were asked to collect the midstream urine sample in a sterile container and were processed in 15–20 min for culture and sensitivity analysis using the disk diffusion method with the VITEK-2 Compact system [22].

A urine culture was considered positive for infection if the colony count for a single organism was more than 10^5^ CFU/mL [23,24]. All isolates were identified and tested for susceptibility by the Vitek 2 system (bioMerieux; Inc., Durham, NC, USA) using the Gram-negative strain cards AST-N291 and Gram-positive strain card AST-P580 [25,26,27]. The isolates were tested for susceptibility for the following antimicrobial agents: amikacin, amoxicillin/clavulanate, ampicillin, cefazolin, cefepime, cefotaxime, cefoxitin, ceftazidime, cefuroxime, ciprofloxacin, ertapenem, gentamicin, imipenem, levofloxacin, meropenem, mezlocillin, moxifloxacin, nitrofurantoin, norfloxacin, piperacillin/tazobactam, piperacillin, tetracycline, tigecycline, tobramycin, co-trimoxazole, and trimethoprim. All results were interpreted using the Advanced Expert System (software version VT2-R04.03). Using the Vitek 2 system AST-N0291 card according to the manufacturer’s recommendations, the isolates were initially considered positive for ESBL if the minimum inhibitory concentration of ceftazidime and cefotaxime for these organisms was ≥2 mg/L.

### Statistical Analysis

Statistical analysis was performed by IBM^®^ SPSS, version 26 (SPSS Inc., Chicago, IL, USA). Person Chi-square (χ^2^) tests were used for analyzing categorical variables and expressed as frequency and percentage. Differences were considered significant at *p* < 0.05.

## 3. Results

Over the one year, a total of 136 culture reports from outpatient urine samples were available. From those, only 123 (90.45%) were found to show significant growth for UTIs. The age of the participants ranged from 18 to 65 years (median: 31 years and mean ± SD = 32.14 ± 9.83). Demographic data for the age of patients age is given in Table 1. The most commonly isolated organism was *E. coli* (58.5%), followed by *K. pneumoniae* (8.1%), as shown in Table 2. Stratified by age, the most commonly isolated uropathogens in women aged ≥45 years and those <45 years were *E. coli* (90.27% and 9.73% and *Klebsiella* (100% and 0.0%), respectively. All other uropathogens, except for *Enterobacter cloacae* (50%), *E. coli* (9.7%), and *Sphingomonas paucimobilis* (*S. paucimobilis*) (20 %), were isolated only from women ≥45 years of age.

No significant difference was found between age groups to distribute isolated uropathogens (*p* = 0.980).

**Figure 1:**
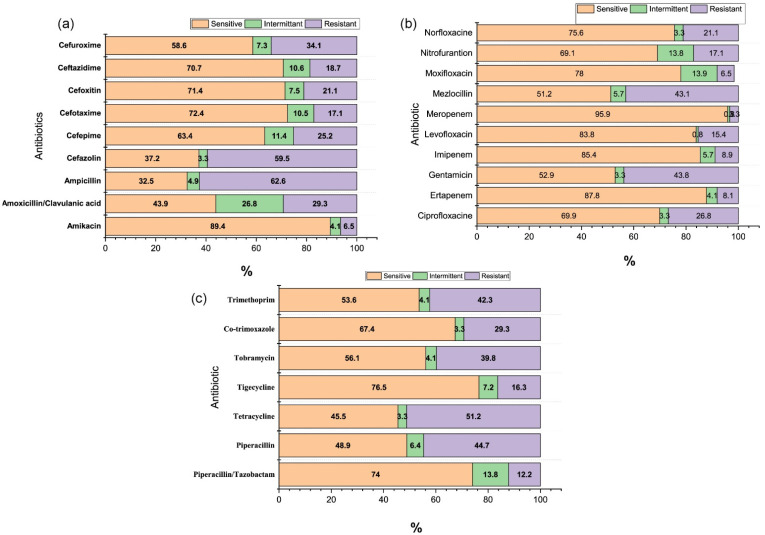
(**a**–**c**) Overall sensitivity, and intermittent and resistant pattern, of isolated uropathogens to the tested antibiotics.

In our study, all isolated uropathogens, except *Streptococcus agalactiae* (*S. agalactiae*)*, Staphylococcus hominis* (*S. hominis*), and *Enterobacter amnigenus* (*E. amnigenus*), showed increased resistance to a wide range of used antibiotics, including recently produced and expensive ones. Overall, the rate of antibiotic resistance was highest for ampicillin (62.6%), cephazolin (59.5%), tetracycline (51.2%), gentamycin (43.8%), mezlocillin (43.1%), piperacillin (44.7%), trimethoprim (42.3%), tobramycin (39.8%), and co-trimoxazole (29.3%) (*p* = 0.053). Among *E. coli* isolates, 56.94% were resistant to ampicillin, 52.8% to co-trimoxazole, 48.6% to Mezlocillin, 45.8% to piperacillin, 44.4% to trimethoprim, and 34.7% to cefazolin. Furthermore, amikacin, ertapenem, and nitrofurantoin demonstrated a significantly high susceptibility pattern (0% resistance) for *E. coli* (Figure S1). Additionally, the antibiotic sensitivity pattern for *E. coli* against ciprofloxacin, levofloxacin, moxifloxacin imipenem, meropenem, norfloxacin, and piperacillin/tazobactam was more than 90%. ESBL-producing strains in urinary isolates of *E. coli* were 6.5% *K. pneumoniae* 0.81%, *K. oxytoca* 0.81%, and *Citrobacter amalonaticus (C. amalonaticus)* 0.81%. More than 90% of the ESBL producers were susceptible to imipenem and meropenem. The resistance pattern of all the isolated uropathogens with tested antibiotics is shown in Table 3, Table 4 and Table 5, and overall sensitivity, and intermittent and resistant patterns, of isolated uropathogens to the tested antibiotics are shown in the Figure 1a–c. The obtained results were statistically not significant between isolated uropathogens and the pattern of resistance (*p* = 0.255).

## 4. Discussion

Antimicrobial drug resistance is considered a serious global challenge. The leading causes of bacterial gene mutations and eventual resistance to antibiotics are an inappropriate use of broad-spectrum antibiotics, excessive prescribing of broad-spectrum antibiotics by physicians, and improper use of antibiotics by patients [25]. Various local studies reported the high prevalence of antimicrobial resistance and ESBL strains in different regions of Saudi Arabia [12,16,25,28,29,30]. For example, an investigation by Kader and colleagues [12] revealed that ESBL-producing strains showed the highest susceptibility to imipenem and meropenem (96%).

The present study reports the antimicrobial resistance patterns of uropathogens isolated from females patients in the Najran region of Saudi Arabia. Most studies have identified *E. coli* as a leading causative agent of UTIs worldwide [7,9,30,31,32,33]. Our reports confirmed that *E. coli* (58.5%) was the most common causative organism of UTIs, followed by *K. pneumoniae* (8.1%). Furthermore, the resistance rate to ampicillin among *E. coli* isolates was 56.94%. This is lower than the previous study in another region of Saudi Arabia [25,34]. Additionally, in our study, the overall resistance rate to ampicillin among isolated uropathogens was 62.6%, comparable to four studies from Africa and four from Asia (67.2%) [31]. Our results indicated a high resistance (44.4% and 48.6%, respectively) for trimethoprim and mezlocillin resistance against *E. coli*. The rate of co-trimoxazole resistance to *E. coli* was 52.8% and is comparable with previous studies in another region of Saudi Arabia [25,35].

Our results are consistent with a recent study that sought to determine the antimicrobial resistance patterns among different *E. coli* isolates in the city of Riyadh [36]. The isolates from the human urine samples were most frequently resistant to norfloxacin (80%), followed by amoxicillin and ampicillin (70%); trimethoprim/sulfamethoxazole (55%); ciprofloxacin and ofloxacin (50%); cefalotin (30%); cefuroxime, cefixime, and cefotaxime (25%); ceftazidime, ceftriaxone, cefepime, and aztreonam (20%); amoxicillin/clavulanic acid, piperacillin/tazobactam, and gentamicin (10%); and amoxicillin/sulbactam and cefoxitin (5%) [36]. Furthermore, 95.8% of the isolates were antimicrobial-resistant, and 16.7% were positive for ESBL [36]. Similarly, Bandy et al. reported the high prevalence of antimicrobial resistance *Enterobacterales* in the Aljouf region of Saudi Arabia [37]. Another study by Amari et al. reported the high prevalence of *E. coli* (33.7%), *K. pneumoniae*, 15.21%, *P. aeruginosa* (10.2%), and *A. baumannii* (5.3%) from the urine samples of patients in the Asir region of Saudi Arabia [38]. Furthermore, a high resistance rate was observed against ampicillin for *E. coli* (88.3%), *K. pneumoniae* (93.7%), *P. aeruginosa* (98.9%), and *A. baumannii* (97.9%), respectively [38]. However, this is considered very high compared with other studies in Canada (11%) and United States (16–18%) [39]. The explanation for this difference might be that these antibiotics can be bought easily from the community pharmacy in our country compared to the strict restrictions imposed in other countries and misuse of antibiotics in diseases such as cold, flu, some ear infection, and cough in our country. Our finding of increased resistance to ampicillin, trimethoprim, mezlocillin, and co-trimoxazole suggests that these agents may no longer be suitable for empiric therapy.

From our study, fluoroquinolones and nitrofurantoin have been suggested as appropriate empiric treatments for UTIs. This might be reasonable with a resistance rate of 6.9% for ciprofloxacin, 4.2% for levofloxacin, 5.6% for norfloxacin, and 0% nitrofurantoin against E. coli. However, more extensive use of this antibiotic may raise the alarms of increased resistance patterns in the near future. The isolated *E. coli* strains were most susceptible to amikacin, cefepime, ertapenem, imipenem, meropenem, and tigecycline. This may be due to the limited availability and high cost of these drugs in our country. *Staphylococcus saprophyticus* is a well-known urinary pathogen in acute cystitis in young females. However, it was not present in any of the reports obtained from the hospital laboratory.

This report will significantly impact UTIs treatment in the Najran region of Saudi Arabia. However, there is a certain limitation associated with our study. For example, the population is limited to one particular region of Saudi Arabia. This study was performed in Najran city of Saudi Arabia, and this does not necessarily reflect the antimicrobial resistance trends in another region within the same country. A larger cohort of people from a different region of Saudi Arabia with a different health condition may offer more information. Additionally, the investigation results were based on the culture reports from three different microbiology laboratories, which might have variations in resistance rate due to different procedures or any other reason. Furthermore, side effects/adverse effects associated with each antibiotic were not reported.

## 5. Conclusions

Our study reported the prevalence of *E. coli* as a significant uropathogens among female patients in the Najran region of Saudi Arabia. This study provides essential information about the antimicrobial susceptibility patterns in uropathogens from the Najran Region in Saudi Arabia and reveals the prevalence of ESBL production in the region. The obtained results emphasize that *E. coli* remains the most commonly isolated uropathogens, followed by *Klebsiella* species. Isolated uropathogens demonstrated high sensitivity to amikacin, cefepime, ertapenem, imipenem, meropenem, and tigecycline. It is always necessary to have an understanding of the bacterial species and their antimicrobial sensitivity patterns in order to serve as a basis for selecting the empirical treatment of urinary tract infections (UTIs) as resistance rates vary geographically.

## Figures and Tables

**Table 1:** Demographic data of study population.

Age Group	% Frequency (*n* = 123)
18–30	48.8 (60)
31–40	35.8 (44)
41–50	8.9 (11)
Above 50	6.5 (8)

**Table 2:** Microorganisms isolated from patients of different age groups.

Microorganism (%)	Total *n* = 123	Age Group					
		18–25	26–30	31–35	36–40	41–45	Above 45
*Acinetobacter baumannii*	4 (3.3)	2 (50)	2 (50)	0 (0)	0 (0)	0 (0)	0 (0)
*Citrobacter amalonaticus*	1 (0.8)	1 (100)	0 (0)	0 (0)	0 (0)	0 (0)	0 (0)
*Enterobacter aerogenes*	1 (0.8)	0 (0)	0 (0)	1 (100)	0 (0)	0 (0)	0 (0)
*Enterobacter amnigenus*	1 (0.8)	0 (0)	0 (0)	0 (0)	1 (100)	0 (0)	0 (0)
*Empedobacter brevis*	1 (0.8)	0 (0)	1 (100)	0 (0)	0 (0)	1 (100)	0 (0)
*Enterobacter cloacae*	4 (3.3)	0 (0)	0 ((0)	0 (0)	1 (25%)	1 (25)	2 (50)
*Escherichia coli*	72 (58.5)	14 (19.4)	18 (25)	16 (22.2)	11 (15.3)	6 (8.3)	7 (9.7)
*Klebsiella oxytoca*	1 (0.8)	0 (0)	0 (0)	0 (0)	1 (100)	0 (0)	0 (0)
*Klebsiella pneumoniae*	10 (8.1)	2 (20)	5 (50)	2 (20)	1 (10)	0 (0)	0 (0)
*Kocuria kristinae*	2 (1.6)	1 (50)	0 (0)	0 (0)	1 (50)	0 (0)	0 (0)
*Proteus mirabilis*	1 (0.8)	1 (100)	0 (0)	0 (0)	0 (0)	0 (0)	0 (0)
*Providencia rettgeri*	1 (0.8)	0 (0)	1 (100)	0 (0)	0 (0)	0 (0)	0 (0)
*Providencia stuartii*	3 (2.4)	3 (100)	0 (0)	0 (0)	0 (0)	0 (0)	0 (0)
*Pseudomonas aeruginosa*	2 (1.6)	1 (50)	0 (0)	1 (50)	0 (0)	0 (0)	0 (0)
*Pseudomonas oryzihabitans*	1 (0.8)	1 (100)	0 (0)	0 (0)	0 (0)	0 (0)	0 (0)
*Salmonella sp*	2 (1.6)	1 (50)	0 (0)	1 (50)	0 (0)	0 (0)	0 (0)
*Serratia marcescens*	2 (1.6)	0 (0)	2 (100)	0 (0)	0 (0)	0 (0)	0 (0)
*Sphingomonas paucimobilis*	5 (4.1)	2 (40)	0 (0)	1 (20)	0 (0)	1 (20)	1 (20)
*Staphylococcus epidermidis*	5 (4.1)	1 (20)	0 (0)	3 (60)	0 (0)	1 (20)	0 (0)
*Staphylococcus haemolyticus*	2 (1.6)	1 (50)	1 (50)	0 (0)	0 (0)	0 (0)	0 (0)
*Staphylococcus hominis*	1 (0.8)	0 (0)	1 (100)	0 (0)	0 (0)	0 (0)	0 (0)
*Streptococcus agalactiae*	1 (0.8)	0 (0)	1 (100)	0 (0)	0 (0)	0 (0)	0 (0)

**Table 3:** Distribution of resistance of isolated uropathogens to tested antimicrobials.

Microorganism (%)	Amikacin	Amox + Clav	Ampicillin	Cefazolin	Cefepime	Cefotaxime	Cefoxitin	Ceftazidime	Cefuroxime
*Acinetobacter baumannii*	1 (25)	3 (75)	3 (75)	3 (75)	0 (0)	0 (0)	2 (50)	1 (25)	2 (50)
*Citrobacter amalonaticus*	0 (0)	1 (100)	1 (100)	1 (100)	1 (100)	0 (0)	1 (100)	0 (0)	1 (100)
*Enterobacter aerogenes*	1 (100)	0 (0)	1 (100)	1 (100)	1 (100)	1 (100)	1 (100)	1 (100)	1 (100)
*Enterobacter amnigenus*	0 (0)	0 (0)	0 (0)	1 (100)	0 (0)	0 (0)	1 (100)	0 (0)	0 (0)
*Empedobacter brevis*	0 (0)	0 (0)	0 (0)	0 (0)	0 (0)	0 (0)	0 (0)	0 (0)	0 (0)
*Enterobacter cloacae*	0 (0)	2 (50)	3 (75)	3 (75)	1(25)	1 (25)	2 (50)	2 (50)	4 (100)
*Escherichia coli*	0 (0)	9 (12.5)	41 (59.6)	25 (34.7)	16 (22.2)	4 (5.6)	5 (6.9)	4 (5.6)	17 (23.6)
*Klebsiella oxytoca*	0 (0)	0 (0)	1 (100)	1 (100)	1 (100)	1 (100)	0 (0)	0 (0)	1 (100)
*Klebsiella pneumoniae*	0 (0)	3 (30)	9 (90)	4 (40)	0 (0)	1 (10)	2 (20)	2 (20)	2 (20)
*Kocuria kristinae*	0 (0)	1 (50)	1 (50)	1 (50)	1 (50)	2 (100)	1 (50)	1 (50)	2 (100)
*Proteus mirabilis*	0 (0)	0 (0)	0 (0)	0 (0)	0 (0)	0 (0)	0 (0.0)	0 (0.0)	0 (0.0)
*Providencia rettgeri*	0 (0.0)	1 (100)	1 (100)	1 (100)	0 (0.0)	0 (0.0)	0 (0)	1 (100)	1 (100)
*Providencia stuartii*	1 (33.3)	3 (100)	2 (66.7)	2 (66.7)	1 (33.3)	1 (33.3)	2 (66.7)	0 (0)	2 (66,7)
*Pseudomonas aeruginosa*	0 (0)	2 (100)	2(100)	2 (100)	2 (100)	2(100)	2(100)	2(100)	2 (100)
*Pseudomonas oryzihabitans*	1 (100)	1 (100)	0 (0)	0 (0)	0 (0)	1 (100)	1 (100)	0 (0)	0 (0)
*Salmonella* *sp*	1 (50)	2 (100)	2 (100)	2 (100)	2 (100)	2 (100)	2 (100)	1 (50)	1 (50)
*Serratia marcescens*	1 (50)	1 (50%)	2 (100)	2 (100)	1 (50)	1 (50)	0 (0)	2 (100)	2 (100)
*Sphingomonas paucimobilis*	1 (20)	3 (60%)	3 (60)	1 (20)	1 (20)	1 (20)	1 (20)	2 (40)	1 (20)
*Staphylococcus epidermidis*	1 (20)	2 (40%)	2 (40)	2 (40)	1 (20)	1 (20)	1 (20)	2 (40)	1 (20)
*Staphylococcus haemolyticus*	2 (100)	2 (100)	2 (100)	2 (100)	2 (100)	2 (100)	2 (100)	2 (100)	2 (100)
*Staphylococcus hominis*	0 (0)	0 (0)	1 (100)	0 (0)	0 (0)	0 (0)	0 (0)	0 (0)	0 (0)
*Streptococcus agalactiae*	0 (0)	0 (0)	0 (0)	0 (0)	0 (0)	0 (0)	0 (0)	0 (0)	0 (0)

**Table 4:** Distribution of resistance of isolated uropathogens to tested antimicrobials.

Microorganism (%)	Ciprofloxacin	Ertapenem	Gentamicin	Imipenem	Levofloxacin	Meropenem	Mezlocillin	Moxifloxacin	Nitrofurantoin
*Acinetobacter baumannii*	3 (75)	2 (50)	3 (75)	1 (25)	2 (50)	2 (50)	2 (50)	0 (0)	2 (50)
*Citrobacter amalonaticus*	1 (100)	0 (0)	0 (0)	0 (0)	1 (100)	0 (0)	1 (100)	0 (0)	1 (100)
*Enterobacter aerogenes*	1 (100)	1 (100)	0 (0)	0 (0)	0 (0)	0 (0)	1 (100)	0 (0)	0 (0)
*Enterobacter amnigenus*	0 (0)	0 (0)	0 (0)	0 (0)	0 (0)	0 (0)	0 (0)	0 (0)	1 (100)
*Empedobacter brevis*	0 (0)	0 (0)	0 (0)	0 (0)	0 (0)	0 (0)	0(0)	0 (0)	0 (0)
*Enterobacter cloacae*	3 (75)	1 (25)	0 (0)	0 (0)	2 (50)	0 (0)	4 (100)	1 (25)	2 (50)
*Escherichia coli*	5 (6.9)	0 (0)	13 (18.1)	2 (2.8)	3 (4.2)	1 (1.4)	35 (48.6)	3 (4.2)	0 (0)
*Klebsiella oxytoca*	1 (100)	0 (0)	0 (0)	0 (0)	1 (100)	0 (0)	1 (100)	0 (0)	0 (0)
*Klebsiella pneumoniae*	4 (40)	0 (0)	2 (20)	0 (0)	0 (0)	0 (0)	7 (70)	0 (0)	1 (10)
*Kocuria kristinae*	1 (50)	1 (50)	2 (100)	1 (50)	2 (100)	0 (0)	1 (50)	1 (50)	1 (50)
*Proteus mirabilis*	0 (0)	0 (0)	0 (0)	0 (0)	0 (0)	0 (0)	0 (0)	1 (100)	1 (100)
*Providencia rettgeri*	0 (0)	0 (0)	1 (100)	1 (100)	0 (0)	0 (0)	1 (100)	0 (0)	0 (0)
*Providencia stuartii*	3 (100)	1 (33.3)	3 (100)	0 (0)	2 (66.6)	1 (33.3)	0 (0)	0 (0)	3 (100)
*Pseudomonas aeruginosa*	2(100)	0 (0)	2 (100)	0 (0)	0 (0)	0 (0)	0 (0)	0 (0)	2 (100)
*Pseudomonas oryzihabitans*	1 (100)	0 (0)	1 (100)	0 (0)	0 (0)	0 (0)	0 (0)	0 (0)	1 (100)
*Salmonella sp*	2 (100)	1 (50)	2 (100)	1 (50)	2 (100)	0 (0)	1 (50)	0 (0)	0 (0)
*Serratia marcescens*	0 (0)	0 (0)	1 (50)	2 (100)	1 (50)	0 (0)	0 (0)	1 (50)	0 (0)
*Sphingomonas paucimobilis*	2 (40)	1 (20)	2 (40)	1 (20)	1 (20)	0 (0)	0 (0)	1 (20)	2 (40)
*Staphylococcus epidermidis*	2 (40)	0 (0)	2 (40)	0 (0)	0 (0)	0 (0)	0 (0)	0 (0)	1 (20)
*Staphylococcus haemolyticus*	2 (100)	2 (100)	2 (100)	2 (100)	2 (100)	0 (0)	0 (0)	0 (0)	2 (100)
*Staphylococcus hominis*	0 (0)	0 (0)	0 (100)	0 (0)	0 (0)	0 (0)	0 (0)	0 (0)	0 (0)
*Streptococcus agalactiae*	0 (0)	0 (0)	0 (0)	0 (0)	0 (0)	0 (0)	0 (0)	0 (0)	1 (100)

**Table 5:** Distribution of resistance of isolated uropathogens to tested antimicrobials.

Microorganism (%)	Norfloxacin	Piperacillin-Tazobactam	Piperacillin	Tetracycline	Tigecycline	Tobramycin	Co-trimoxazole	Trimethoprim
*Acinetobacter baumannii*	2 (50)	3 (75)	2 (50)	2 (50)	2 (50)	2 (50)	3 (75)	3 (75)
*Citrobacter amalonaticus*	1 (100)	0 (0)	1 (100)	1 (100)	0 (0)	0 (0)	1 (100)	1 (100)
*Enterobacter aerogenes*	0 (0)	0 (0)	1 (100)	1 (100)	0 (0)	0 (0)	1 (100)	1 (100)
*Enterobacter amnigenus*	0 (0)	0 (0)	0 (0)	0 (0)	0 (0)	0 (0	1 (100)	0 (0)
*Empedobacter brevis*	0 (0)	0 (0)	0 (0)	0 (0)	0 (0)	1 (100)	1 (100)	0 (0)
*Enterobacter cloacae*	2 (50)	0 (0)	3 (75)	4 (100)	0 (0)	2 (50)	3 (75)	2 (50)
*Escherichia coli*	4 (5.6)	7 (9.7))	33 (45.8)	35 (48.6)	5 (6.9)	19 (26.4)	38 (52.8)	32 (44.4)
*Klebsiella oxytoca*	1 (100)	0 (0)	1 (100)	1 (100)	0 (0)	0 (0)	1 (100)	1 (100)
*Klebsiella pneumoniae*	3 (30)	1 (10)	7 (70)	4 (40)	2 (20)	5 (50)	5 (50)	4 (40)
*Kocuria kristinae*	2 (100)	0 (0)	0 (0)	2 (100)	1 (50)	2 (100)	1 (50)	0 (0)
*Proteus mirabilis*	0 (0)	0 (0)	0 (0	0 (0)	0 (0)	0 (0)	0 (0)	0 (0)
*Providencia rettgeri*	0 (0)	0 (0)	1 (100)	1 (100)	1 (100)	1 (100)	1 (100)	1 (100)
*Providencia stuartii*	3 (100)	2 (66.7)	1 (33.3)	3 (100)	1 (33.3)	2 (66.7)	3 (100)	1 (33.3)
*Pseudomonas aeruginosa*	2 (100)	0 (0)	0 (0)	0 (0)	2 (100)	2 (100)	2 (100)	0 (0)
*Pseudomonas oryzihabitans*	1 (100)	1 (100)	0 (0)	1 (100)	0 (0)	0 (0)	1 (100)	0 (0)
*Salmonella sp*	1 (50)	0 (0)	0 (0)	2 (100)	1 (50)	2 (100)	2 (100)	0 (0)
*Serratia marcescens*	0 (0)	1 (50)	2 (100)	1 (50)	1 (50)	2 (100)	2 (100)	2 (100)
*Sphingomonas paucimobilis*	1 (20)	0 (0)	1 (20)	2 (40)	1 (20)	2 (40)	2 (40)	1 (20)
*Staphylococcus epidermidis*	1 (20)	0 (0)	0 (0)	0 (0)	1 (20)	4 (80)	5 (100)	1 (20)
*Staphylococcus haemolyticus*	2 (100)	0 (0)	2 (100)	2 (100)	2 (100)	2 (100)	2 (100)	2 (100)
*Staphylococcus hominis*	0 (0)	0 (0)	0 (0)	1 (100)	0 (0)	1 (100)	0 (0)	0 (0)
*Streptococcus agalactiae*	0 (0)	0 (0)	0 (0)	0 (0)	0 (0)	0 (0)	0 (0)	0 (0)

## Supplementary Materials

The following are available online at https://www.mdpi.com/article/10.3390/clinpract11030080/s1, Figure S1: Overall sensitivity, and intermittent and resistant pattern, of isolated *E. coli* the tested antibiotics
