# Supplementary material for: Antimicrobial Resistance Patterns and ESBL of Uropathogens Isolated from Adult Females in Najran Region of Saudi Arabia
Source: Clin Pract. 2021 Sep 14;11(3):650–8. doi: 10.3390/clinpract11030080 (PMC8482141; doi:10.3390/clinpract11030080)
Supplement: Supplementary file 1 [file clinpract-11-00080-s001.zip › clinpract-1308454-supplementary.pdf]

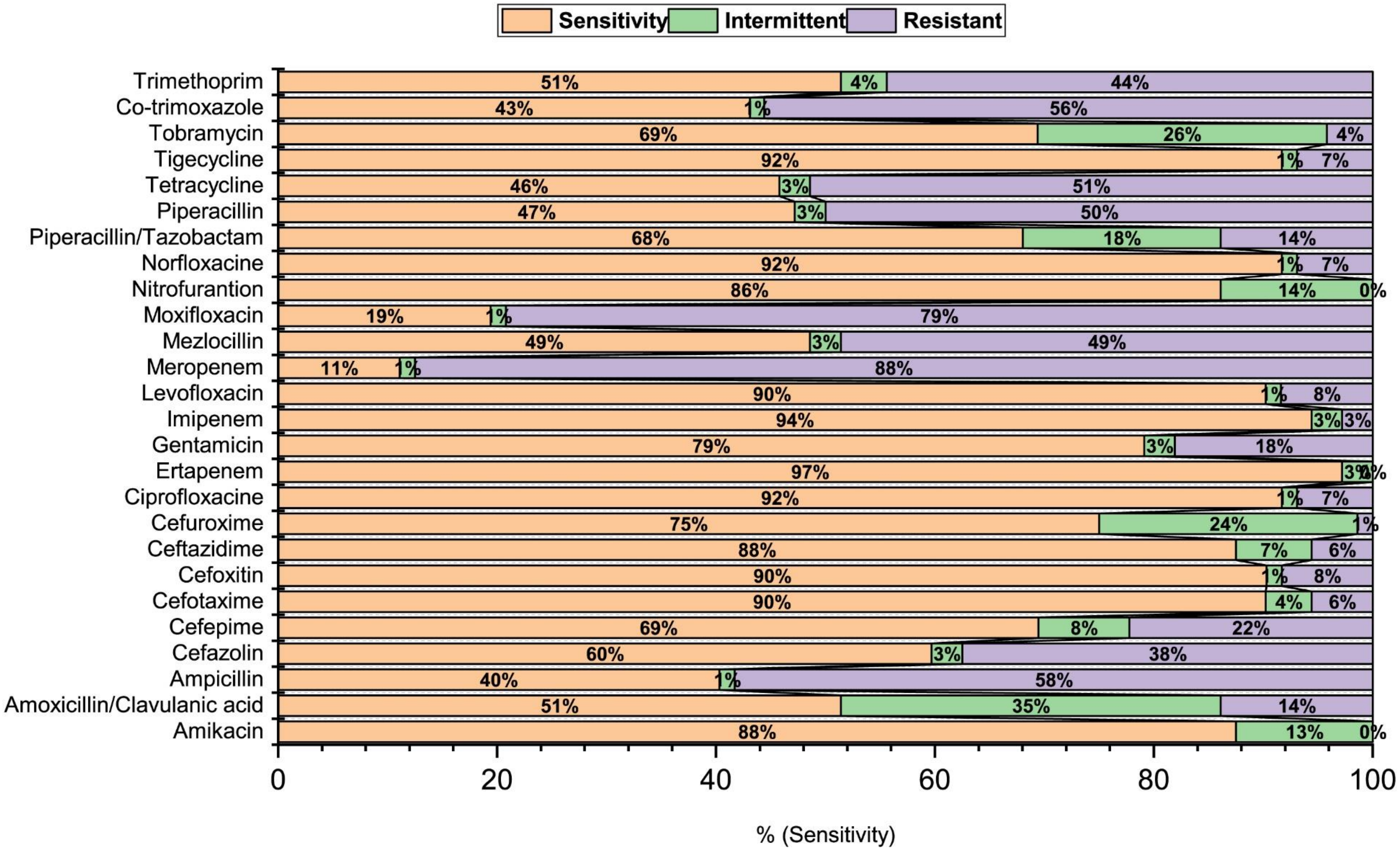

Figure S1. Overall sensitivity, and intermittent and resistant pattern, of isolated *E. coli* the tested antibiotics
